# Efficient Isolation of Pure and Functional Mitochondria from Mouse Tissues Using Automated Tissue Disruption and Enrichment with Anti-TOM22 Magnetic Beads

**DOI:** 10.1371/journal.pone.0082392

**Published:** 2013-12-12

**Authors:** Andras Franko, Olivier R. Baris, Eva Bergschneider, Christine von Toerne, Stefanie M. Hauck, Michaela Aichler, Axel K. Walch, Wolfgang Wurst, Rudolf J. Wiesner, Ian C. D. Johnston, Martin Hrabĕ de Angelis

**Affiliations:** 1 Institute of Experimental Genetics, Helmholtz Zentrum München, German Research Center for Environmental Health, Neuherberg, Germany; 2 Institute of Vegetative Physiology, Medical Faculty, University of Köln, Köln, Germany; 3 Miltenyi Biotec GmbH, Bergisch Gladbach, Germany; 4 Research Unit Protein Science, Helmholtz Zentrum München, German Research Center for Environmental Health, Neuherberg, Germany; 5 Research Unit Analytical Pathology, Institute of Pathology, Helmholtz Zentrum München, German Research Center for Environmental Health, Neuherberg, Germany; 6 Institute of Developmental Genetics, Helmholtz Zentrum München, Technische Universität München, Neuherberg, Germany; 7 Max Planck Institute of Psychiatry, Munich, Germany; 8 Technische Universität München, Lehrstuhl für Entwicklungsgenetik, c/o Helmholtz Zentrum München, Neuherberg, Germany; 9 DZNE – Deutsches Zentrum fuer Neurodegenerative Erkrankungen, Site Munich, Germany; 10 Center for Molecular Medicine (CMMC), University of Köln, Köln, Germany; 11 Cologne Excellence Cluster on Cellular Stress Responses in Ageing-associated Diseases (CECAD), University of Köln, Köln, Germany; 12 Technische Universität München, WZW - Center of Life and Food Science Weihenstephan, Chair of Experimental Genetics, Freising-Weihenstephan, Germany; 13 German Center for Diabetes Research (DZD), Neuherberg, Germany; University of Iowa, United States of America

## Abstract

To better understand molecular mechanisms regulating changes in metabolism, as observed e.g. in diabetes or neuronal disorders, the function of mitochondria needs to be precisely determined. The usual isolation methods such as differential centrifugation result in isolates of highly variable quality and quantity. To fulfill the need of a reproducible isolation method from solid tissues, which is suitable to handle parallel samples simultaneously, we developed a protocol based on anti-TOM22 (translocase of outer mitochondrial membrane 22 homolog) antibody-coupled magnetic beads. To measure oxygen consumption rate in isolated mitochondria from various mouse tissues, a traditional Clark electrode and the high-throughput XF Extracellular Flux Analyzer were used. Furthermore, Western blots, transmission electron microscopic and proteomic studies were performed to analyze the purity and integrity of the mitochondrial preparations. Mitochondrial fractions isolated from liver, brain and skeletal muscle by anti-TOM22 magnetic beads showed oxygen consumption capacities comparable to previously reported values and little contamination with other organelles. The purity and quality of isolated mitochondria using anti-TOM22 magnetic beads was compared to traditional differential centrifugation protocol in liver and the results indicated an obvious advantage of the magnetic beads method compared to the traditional differential centrifugation technique.

## Introduction

Mitochondrial performance needs to be investigated in many human disorders, as their altered function often contributes to or is at least suspected to play a role in the development of disease [1]. For example, mitochondrial dysfunction in neuronal tissues has been associated with epilepsy [2] as well as neurodegenerative disorders and ageing [3], [4]. Moreover, disturbed mitochondrial function has been observed in skeletal muscle and in liver of patients with type 2 diabetes [5], [6]. An association between mitochondrial dysfunction and insulin-resistance or diabetes has also been described in mice [7], [8]. Therefore, mice appear to be an appropriate model for understanding the molecular mechanisms underlying such disorders, and exploring how mitochondrial function can affect tissues.

Since one of the major functions of mitochondria is the production of ATP via oxidative phosphorylation, measurements of oxygen consumption using isolated organelles are commonly performed to identify potential mitochondrial dysfunction. In recent years, new devices such as the XF Extracellular Flux Analyzer were developed to measure oxygen consumption in many samples simultaneously, including isolated mitochondria, thus allowing the exploration of mitochondrial function in smaller samples and in a highly parallel fashion compared to the traditional Clark electrode [9], [10]. To match the convenience of such high-throughput devices for analyzing mitochondrial respiration in mouse tissues, there is a need for a standardized isolation method that allows simultaneous handling of numerous samples.

Numerous protocols of mitochondria isolation rely on gradient centrifugation steps [11], [12]. The gradient centrifugation (GC) method applies gradient of sucrose or Percoll and fractions are collected at very high speed (usually with ultracentrifugation). The obtained mitochondrial fractions show good purity and in the case of brain tissue the GC method yields in pure mitochondrial fractions, which lack synaptosomal contaminations [11]. On the other hand mitochondrial fraction isolated by GC from mouse liver still contains lysosomal, ER and peroxisomal contaminations [13]. In contrast to gradient centrifugation, differential centrifugation methods are more simple, they do not require ultracentrifugation and can be performed faster [14], [15]. In brief, the tissue is homogenized, cell debris is discarded at low speed and the mitochondria contained in the supernatant are collected in a second centrifugation step at high speed. This differential centrifugation (DC) method is simple, but has several limitations. The homogenization step is crucial, since distinct tissues need distinct homogenization forces to disrupt cells while maintaining the integrity of mitochondrial membranes. For example, less force is required to disrupt liver compared to hard tissues such as muscle. However, in most laboratories, this step is performed manually using Potter homogenizers, thus introducing a highly subjective parameter. As homogenization protocols are also different from lab to lab (rotation speed of the pestle, number of strokes, etc.), it is therefore difficult to reproduce experiments across different research groups [16]. Furthermore, when numerous samples are processed at the same time, the centrifugation steps become increasingly difficult to handle and time-consuming. Another limitation of the DC method comes from the lack of purity of the mitochondrial fractions, since with high speed centrifugations other cell organelles are also sedimented, thus contaminating the preparations [15]. Therefore, our aim was to establish a new protocol, which is reproducible, easy to use and suitable for various mouse tissues but, most importantly, avoids non-mitochondrial contaminations.

Recently, Hornig-Do and colleagues described for the first time the isolation of mitochondria from cultured human cell lines using magnetic beads coupled to anti-TOM22 antibodies [17]. These mitochondrial preparations were pure and demonstrated a functional protein import machinery as well as respiratory chain function. In the present study, we developed this method for isolating pure mitochondria from various mouse tissues, which have an adequate quality for further studies. Using our newly developed protocol, we first isolated mitochondria from heart, brain and liver, and checked the purity of the magnetically labeled (elution) and non-labeled (wash) fractions of the anti-TOM22 magnetic beads separations via Western blot experiments. The integrity of isolated mitochondria was analyzed in details by transmission electron microscopy. Furthermore, we studied the functionality and integrity of freshly isolated muscle, liver and brain mitochondria by determining their oxygen consumption rates using a traditional Clarke electrode. Last, to assess whether the new magnetic beads technique has advantages compared to the commonly used differential centrifugation, we extracted mitochondria from liver using both methods, and extensively analyzed the purity and functionality of the isolates via a quantitative proteomic approaches and high-throughput respiration measurements.

## Materials and Methods

### Isolation of Mitochondria

Chemicals and reagents were purchased from Sigma-Aldrich (Taufkirchen, Germany) unless otherwise indicated. All animals used in our study received humane care and since no procedures were performed using live animals and since they were sacrificed (by CO_2_ inhalation) for scientific purposes, according to German Animal Welfare Act (§7, §8 and §8a) no approval were needed for our study. Furthermore, animals were declared to the responsible local authority (Regierung von Oberbayern) and the Institutional Animal Welfare Officer was consulted about the work and approved the study.

#### Differential centrifugation (DC) method

The protocol was performed according to [10], [14]. Briefly, 500 mg liver tissue was washed and minced in PBS supplemented with 10 mM EDTA (PE). The tissue was homogenized with five strokes with a PTFE glass homogenizer (Sartorius, Göttingen, Germany) at 550 rpm in MSHEB buffer (210 mM mannitol, 70 mM sucrose, 5 mM HEPES, 1 mM EGTA, 0.5% BSA pH 7.2) supplemented with Halt protease inhibitor cocktail (Thermo Fisher Scientific, Rockford, IL, USA). The lysate was centrifuged at 1000×g for 5 min at 4°C. Fat was decanted from the supernatant and the lysate was centrifuged at 12,000×g for 10 min at 4°C; this step was performed twice. The final mitochondrial pellet was washed in MSHEB buffer and after another similar centrifugation step, was resuspended in a minimal volume of MSHEB buffer.

#### Anti-TOM22 magnetic beads (MB) isolation method

Tissue lysates were prepared using the Mitochondria Extraction Kit, tissue (Miltenyi Biotec, Bergisch Gladbach, Germany) according to manufacturer’s instructions. 100–150 mg liver, heart, brain and skeletal muscle (quadriceps) tissues were cut into small pieces and digested with Extraction Buffer for 30 min at 4°C. After a brief centrifugation step at 300×g for 5 min at 4°C, the pellet was resuspended in buffer supplemented with Halt protease inhibitor cocktail (Thermo Fisher Scientific) and was quickly homogenized in C-Tubes using the GentleMACS Dissociator (Miltenyi Biotec) with the m_mito_tissue_01 program. The lysate was filtered through a 70 µm pre-separation filter (Miltenyi Biotec) before mitochondria were magnetically enriched with the Mitochondria Isolation Kit, mouse tissue (Miltenyi Biotec) according to manufacturer’s instructions. Briefly, mitochondria were labeled with 50 µl of anti-TOM22 MicroBeads in separation buffer for 1 h at 4°C. The suspension was then passed through a 30 µm pre-separation filter (Miltenyi Biotec) and loaded onto an LS column (Miltenyi Biotec), which had been placed in a MACS Separator. After washing the column with separation buffer, it was removed from the MACS Separator and the magnetically labeled mitochondria were eluted with 1.5 ml separation buffer. Before further analysis, the eluate was centrifuged at 12,000 g for 3 min at 4°C. The mitochondrial pellet was resuspended in PE buffer. The isolation procedure of MB method took about 70–80 minutes from tissue homogenization till mitochondrial elution.

Since the XF Extracellular Flux Analyzer requires different physical parameters compared to traditional Clark electrodes and mitochondria have to be centrifuged onto a cell plate, the isolation protocol was further optimized and applied for XF Extracellular Flux measurements, proteomic and transmission electron microscopy studies. Liver, heart, brain and skeletal muscle were minced in PE buffer supplemented with Halt protease inhibitor cocktail (Thermo Fisher Scientific) and they were directly homogenized using the GentleMACS with program B. The later steps were identical to the above mentioned protocol.

Protein concentrations were determined by BCA (Pierce, Rockford, Il, USA) or Bradford (Biorad, Munich, Germany) methods.

### Western Blot Analysis

Isolated mitochondria were dissolved in CelLytic M cell lysis reagent and 5 µg of mitochondrial and cytosolic proteins were separated using ProGel Tris-Glycine gels (Anamed, Groß-Bieberau, Germany) and transferred to a PVDF membrane for immunoblot analysis. PVDF membranes (Immobilon-P, Millipore, Billerica, MA, USA) were blocked for 1 hour at room temperature with 5% nonfat dry milk, 0.1% Tween-20, in phosphate buffered saline (PBS-T). Primary antibodies and the horseradish peroxidase conjugated secondary antibodies (goat-anti-mouse) were also diluted in PBS-T containing 5% nonfat dry milk. Unbound primary and secondary antibodies were removed by PBS-T washes. The proteins of interest were visualized using a chemiluminescent HRP Substrate (Immobilon Chemiluminescent HRP Substrate, Millipore) according to the manufacturer’s instructions and were detected using photographic film. Antibodies against cytochrome c and PEX1 were purchased from Becton Dickinson (Heidelberg, Germany), TOM20 from Abnova (Taipei, Taiwan) and PDI (protein disulfide isomerase A2) from Santa Cruz (Santa Cruz, CA, USA), the secondary antibody was purchased from Dianova (Hamburg, Germany).

### Measurement of Citrate Synthase (CS) Activity

CS activity was determined by Citrate synthase assay kit (Sigma, Munich, Germany) according to the manual of distributor and samples were measured by Spectrophotometer (SpectraMAX 190 microplate reader, Molecular Devices, Hayward, Ca, USA).

### Transmission Electron Microscopy

Pellets of isolated mitochondrial were fixed in 2.5% electron microscopy grade glutaraldehyde in 0.1 M sodium cacodylate buffer pH 7.4 (Science Services, Munich, Germany), postfixed in 2% aqueous osmium tetraoxide (Dalton, 1955), dehydrated in gradual ethanol (30–100%) and propylene oxide, embedded in Epon (Merck, Darmstadt, Germany) and cured for 24 hours at 60°C. Semithin sections were cut and stained with toluidine blue. Ultrathin sections of 50 nm were collected onto 200 mesh copper grids, stained with uranyl acetate and lead citrate before examination by transmission electron microscopy (Zeiss Libra 120 Plus, Carl Zeiss NTS GmbH, Oberkochen, Germany). Pictures were acquired using Slow Scan CCD-camera and iTEM software (Olympus Soft Imaging Solutions, Münster, Germany).

### Oxygen Consumption Studies Using Clark Electrode

Oxygen consumption studies were performed using a Clark type electrode (Hansatech Instruments, King’s Lynn, England) as described in [18]. 50–150 µg isolated mitochondria were measured in buffer A (300 mM mannitol, 10 mM KH_2_PO_4_, 10 mM KCl, 5 mM MgCl_2_, 1 mg/ml BSA, pH 7.4) at 37°C. Different substrates were used, which feed their electrons into the respiratory chain either via complex I (Final concentrations: 200 µM malate +8 mM pyruvate: MPox; 10 mM malate +10 mM glutamate: MGox), via complex II (5 mM succinate: Sox) or directly into the quinone pool via complex III (5 mM glycero-3-phosphate: GPox). The respiratory control ratio (RCR) was calculated by dividing the rate of succinate oxidation in the presence of the uncoupler carbonyl cyanide 3-chlorophenylhydrazone (2.4 µM CCCP) by the oxidation rate measured previously in the presence of 5 µM oligomycin, an inhibitor of complex V. Mitochondrial respiration was verified by adding 1.6 mM potassium cyanide and no oxygen consumption was detected after this step.

### Oxygen Consumption Studies Using XF Extracellular Flux Analyzer (Seahorse)

After isolation, 2–20 µg mitochondria were plated in MAS-1 buffer (220 mM mannitol, 70 mM sucrose, 10 mM KH_2_PO_4_, 5 mM MgCl_2_, 2 mM HEPES, 1 mM EGTA, 0.2% BSA, pH 7.2) supplemented with succinate (10 mM final concentration) and rotenone (2 µM final concentration) onto a cell plate (Seahorse, North Billerica, MA, USA), which was then centrifuged at 2000×g for 20 min at 4°C. The wells were then filled to 500 µl with MAS-1 succinate+rotenone buffer and incubated at 37°C for 8 minutes. Ports were filled with substrates/inhibitors and the following final concentrations were used in MAS-1 succinate+rotenone buffer: Port A 4 mM ADP, Port B 2 µM oligomycin, Port C 4 µM carbonyl cyanide 4-(trifluoromethoxy)phenylhydrazone (FCCP), Port D 4 µM antimycin A. Succinate was used as a complex II substrate and the plate was analyzed by an XF24 Extracellular Flux Analyzer (Seahorse).

### Liquid Chromatography-Tandem Mass Spectrometry Analysis (LC-MS/MS)

#### Sample preparation

150 µg of each sample were loaded on a 12% gel. After a total separation distance of 1 cm the lanes were fractionated in 2 bands and subjected to in-gel digestion as described in [19]. The supernatants containing the eluted peptides were dried in a speedvac (UniEquip) and stored at −20°C.

#### Mass spectrometry

Prior to the LC-MS/MS analysis, the samples were resuspended in 2% acetonitrile/0.5% TFA and centrifuged for 5 min at 4°C before loading. LC-MS/MS analysis was performed as described previously [20], with the following adjustments: from the MS prescan, the 10 most abundant peptide ions were selected for fragmentation in the linear ion trap if they exceeded an intensity of at least 200 counts and were at least doubly charged. Dynamic exclusion was set to 60 seconds.

#### Non-targeted identification and label-free relative quantification

The RAW files (Thermo Xcalibur file format) were further analyzed using the Progenesis LC-MS software (version 4.1, Nonlinear Dynamics, Newcastle upon Tyne, UK) as described previously [21]. Briefly, the profile data of the MS scans and MS/MS spectra were imported and transformed to peak lists with Progenesis LC-MS using a proprietary algorithm. For peptide identification, Mascot (Matrix Science, version 2.4.1) was set up to search with one missed cleavage allowed, a fragment ion mass tolerance of 0.6 Da and a parent ion tolerance of 10 ppm. Carbamidomethylation was set as fixed modification, methionine oxidation and asparagine or glutamine deamidation were allowed as variable modifications. Spectra were searched against the Ensembl mouse database (Release 72; 51372 sequences) and a Mascot-integrated decoy database. The Mascot Percolator algorithm was used for the discrimination between correct and incorrect spectrum identifications [22] with a cut-off value of 13. The significance threshold of p<0.05 led to a maximum false discovery rate (FDR) of <1%. Peptide assignments were re-imported into Progenesis LC-MS. Proteins with less than 2 peptides used for quantification were excluded. Fold changes were calculated between the averaged abundances of the two conditions for every protein.

### Software Tools and Statistical Analysis

GeneIDs of the MS detected proteins were loaded to Genomatix Software (www.genomatix.de) and were evaluated by Genomatix Pathway System (GePS) tool. Biological Processes GO term was used to filter electron transport chain (ETC) proteins GO:0022900 and Cellular components GO terms were applied for peroxisome GO:0005777, endoplasmic reticulum GO:0005783 and microsome GO:0005792 categories, respectively.

Proteomics data were analyzed by CARMAweb (1.5 release) (https://carmaweb.genome.tugraz.at/carma/). Mann-Whitney U non-parametric tests were applied and *p*-values were adjusted to multiple testing corrections according to Benjamini&Hochberg [23]. Significance level were set to False discovery rate (FDR) <10%.

## Results

### The Anti-TOM22 Magnetic Beads (MB) Method Yields Pure Mitochondrial Fractions from Mouse Tissues

**Figure 1:**
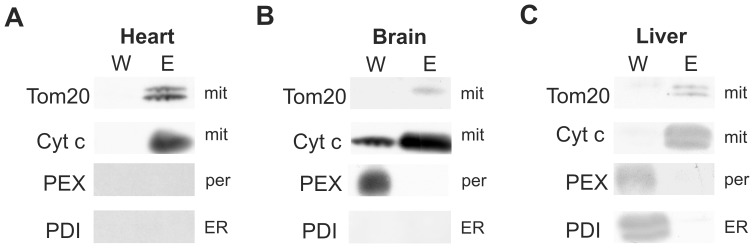
Efficient mitochondrial isolation from mouse tissues using the anti-TOM22 magnetic beads method. Proteins of wash (W) and elution (E) fractions were compared by Western blot analysis from **A** heart, **B** brain and **C** liver tissues. Anti-TOM20 and anti-cytochrome c antibodies were used to detect mitochondrial marker proteins (mit). Anti-PEX1 and PDI antibodies were used to detect peroxisomal (per) or endoplasmic reticulum (ER) marker proteins, respectively.

To test our newly developed protocol, three different mouse tissues, namely heart, brain and liver were chosen and processed by the MB isolation procedure. As a proof of principle, we first compared the wash and elution fractions of the MB isolated mitochondria for the presence of typical organelle markers. Western blots against the mitochondrial markers TOM20 (translocase of outer mitochondrial membrane 20 homolog) and cytochrome c (an inner mitochondrial membrane protein) confirmed the presence of mitochondrial proteins in the elution fractions of all three tissues (Fig. 1). The wash fractions were depleted in the mitochondrial markers with the exception of cytochrome c, which showed a weak signal in the wash fraction from brain (Fig. 1B). Since the common mitochondrial isolation methods do not necessarily selectively discriminate between mitochondria and other organelles such as endoplasmic reticulum (ER) and peroxisomes, we investigated those potential contaminations with organelle-specific antibodies. Western blots were labeled with an antibody against the protein disulfide isomerase A2 (PDI), which is localized in the ER [24], and against PEX1 (peroxisome biogenesis factor 1), a peroxisomal protein. The liver wash fraction showed a strong signal for PDI and PEX1 while these proteins were absent in the mitochondrial fraction (Fig. 1C). The signal for PEX1 protein was again exclusively found in the wash fraction from brain tissue and no signal was detected for PDI in either brain fraction (Fig. 1B). PEX and PDI antibodies did not detect any protein neither in wash nor in elution fractions of heart samples (Fig. 1A). Altogether, these results show that the anti-TOM22 MB method yields pure mitochondrial fractions that are devoid of non-mitochondrial contaminants. In order to assess the enrichment and recovery of the anti-TOM22 MB method, a citrate synthase (CS) assay was performed on crude homogenate and isolated liver mitochondria. When normalized to protein concentration, citrate synthase activity was found to be 6-fold higher in isolated mitochondria (42.8±0.9 µmol/ml/min/mg protein) as compared to the crude homogenate (7.2±0.6 µmol/ml/min/mg protein). Comparison of the total citrate synthase activity in the crude homogenate and the mitochondrial fraction revealed a 19.9±2% recovery. Taken together, these data show that the anti-TOM22 MB technique yields in an enrichment and recovery rate that is comparable to other methods [11], [15].

### Mouse Mitochondria Isolated with Magnetic Beads are Intact and Functional

**Figure 2:**
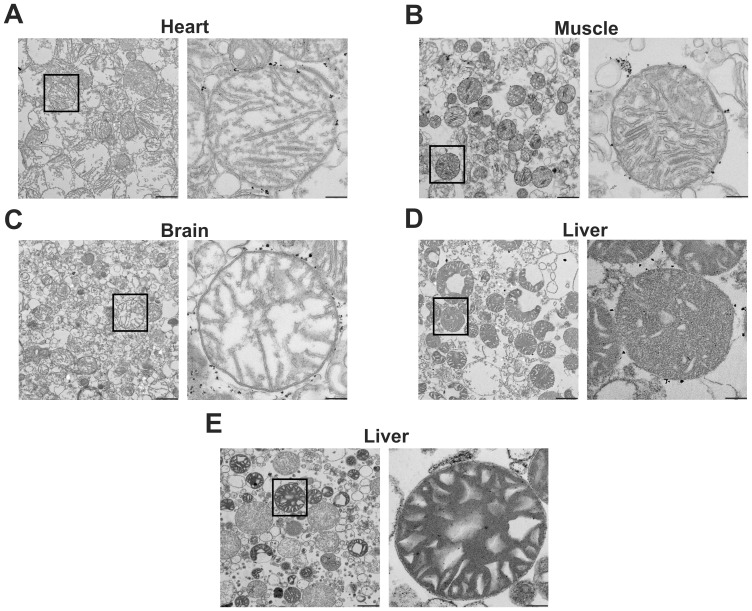
The anti-TOM22 magnetic beads method yields intact mitochondria. Anti-TOM22 magnetic beads method was applied to purify mitochondria from **A** heart, **B** skeletal muscle, **C** brain and **D** liver tissues. Mitochondria were isolated by differential centrifugation technique from **E** liver tissue. Mitochondrial morphology was investigated by transmission electron microscopy from four biological samples per group and representative pictures are shown. Pictures were taken at 4,000× (left panel) and 20,000× (right panel) magnification and black bars display 1 µm (left panel) and 200 nm (right panel), respectively. Black dots at the outer membrane in A, B, C and D pictures represent anti-TOM22 magnetic beads.

**Figure 3:**
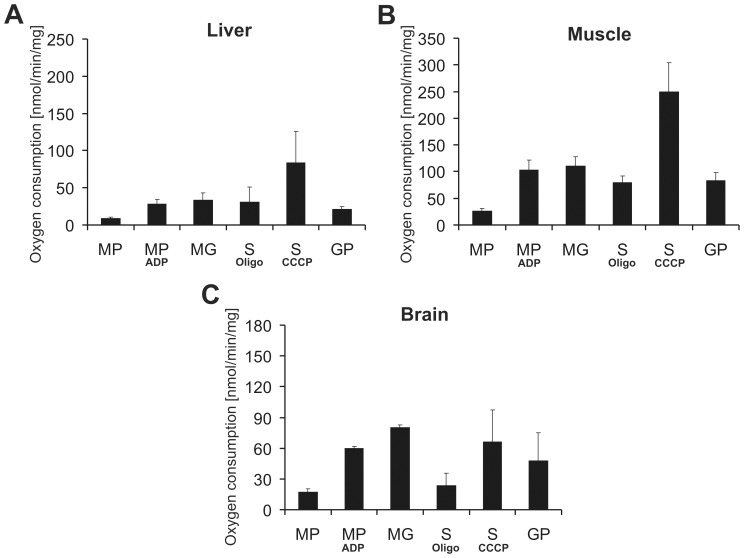
Liver, muscle and brain mitochondria isolated using anti-TOM22 magnetic beads show reliable mitochondrial function. Oxygen consumption of isolated **A** liver, **B** muscle and **C** brain mitochondria were measured by a Clark electrode. Malate+pyruvate (MP) and malate+glutamate (MG) served as specific substrates for complex I, succinate (S, for complex II and glycerol-3-phosphate (GP) for complex III, respectively. ADP was given to malate+pyruvate to determine state III respiration via complex I. Oligomycin and CCCP were used with succinate to assess state IVo and uncoupled respiration, respectively. The given oxygen consumption units are nmol oxygen/min/mg mitochondria. Data are represented as means of three independent experiments ± SD.

To verify the integrity of mitochondria purified with anti-TOM22 magnetic beads, transmission electron microscopic (EM) pictures were taken from isolated heart, skeletal muscle, brain and liver mitochondria (Fig. 2). The morphology of the studied fractions revealed that about 90% of isolated mitochondria were intact. Furthermore EM pictures revealed a good purity of isolated fractions in all four tissues. In order to assess the function of MB isolated mitochondria, we analyzed the oxygen consumption rate using the traditional Clark electrode method. Skeletal muscle and liver as well as brain were chosen as representative hard and soft tissues, respectively. Mitochondria from these tissues showed solid oxygen consumption rates using substrates for complex I (MP, MG), II (S) and III (GP) (Fig. 3). Moreover, to evaluate mitochondrial membrane integrity, respiratory control ratios (RCR) using succinate as a substrate for complex II were determined by dividing the oxygen consumption values obtained after addition of the uncoupler CCCP (uncoupled state) with the values obtained after oligomycin administration (ATPase inhibition; state IVo). The obtained average uncoupled state/state IVo ratios were as follows: for skeletal muscle 3.1±0.5, for liver 3.3±1.5 and for brain 3.0±0.4. According to the oxygen consumption and RCR data as wells as the transmission EM pictures, MB isolated mitochondria displayed intact mitochondrial membranes. Thus, we conclude that automated tissue disruption coupled to MB isolation yields functional and intact mitochondria from both soft and solid mouse tissues.

### Liver Mitochondria Isolated by Magnetic Beads have Higher Purity than those Isolated by Differential Centrifugation

**Figure 4:**
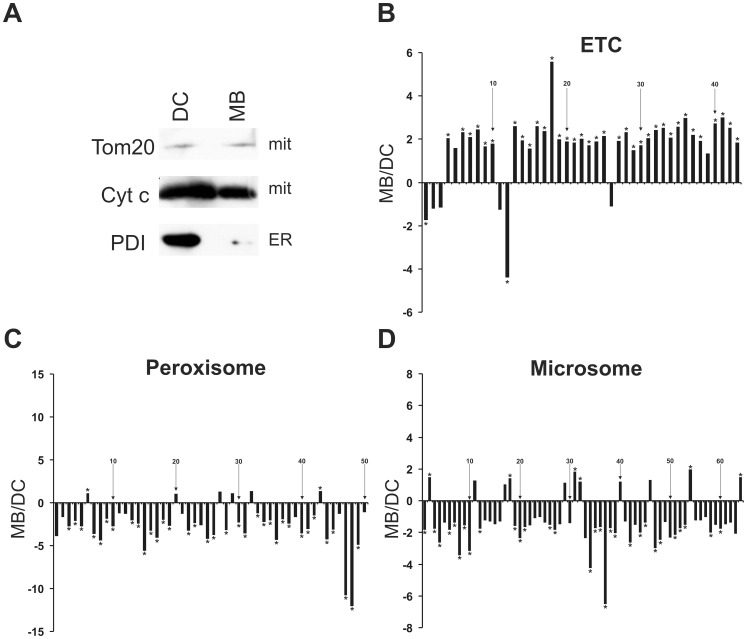
Liver mitochondria isolated by magnetic beads show less non-mitochondrial proteins than mitochondria isolated by differential centrifugation. **A** Mitochondrial preparation obtained by differential centrifugation (DC) was compared to anti-TOM22 magnetic beads (MB) isolation by Western blot using antibodies against mitochondrial (TOM20 and cytochrome c) and ER (PDI) marker proteins. **B–D** Proteins were identified by mass spectrometry and averages of eight samples per group were calculated for MB and DC mitochondrial fractions, respectively. The relative abundance is represented as a linear ratio (average of MB/average of DC). When these ratios were less than 1 (DC average>MB average) the negative inverse ratio was taken and the values were transposed by the equation: −1/(MB/DC ratio). Positive ratios indicate the presence of higher protein levels in the MB fraction, whereas negative ratios indicate higher protein levels in the DC fraction. Diagram titles indicate the applied GO terms, which were identified by Genomatix software GO term analysis. Statistical analyzes were made by Mann-Whitney U test and p values were corrected to multiple testing according to Benjamini&Hochberg [23]. A false discovery rate (FDR) <10% was set as the significance level and significant proteins are denoted by stars. Numbers are given for every tenth protein and the names of the identified proteins are summarized in Table S1. ETC: electron transport chain.

To compare the usual differential centrifugation (DC) to the anti-TOM22 MB method, we first analyzed the presence of TOM20 and Cytochrome c in liver mitochondria isolated with both methods. Western blots showed that, as expected, the two proteins were present in both isolated mitochondrial fractions (Fig. 4A). However, the ER protein marker PDI showed a high abundance in the “mitochondrial” DC fraction, while it gave a very low signal in the MB mitochondrial fraction (Fig. 4A).

Transmission electron microscopic pictures showed intact liver mitochondria in both MB and DC fractions (Fig. 2D–E). To further confirm the results of the Western blot, the purity of DC and MB isolated mitochondrial fractions was analyzed using mass spectrometry (MS) to allow a large-scale and quantitative measurement of their protein content, allowing to discriminate between mitochondrial and non-mitochondrial proteins. Liver mitochondria were isolated from four age-matched animals with the two methods and this analysis was repeated in a second independent experiment. Altogether 702 proteins were identified by MS and these were evaluated according to Cellular Component GO terms by Genomatix software. Out of the 702 identified proteins, 335 (47.7%) belong to the GO term mitochondrion. One should note that the total 1490 mitochondrial proteins belonging to this GO term do not strictly build a closed group. The specialized mitochondrial import machinery plays a key role in transporting proteins into the mitochondria and these proteins are partly unidentified or mostly remain unclassified [25]. Therefore, a mitochondrial protein proportion of 47.7% according to this GO term indicates a significant mitochondrial enrichment. Since the mitochondrion GO term does not exclusively contain mitochondrial proteins, but also well-known cytoplasmic proteins such as superoxide dismutase 1 (Sod1), we decided to use a more accurate group of mitochondrial ontology and applied the Biological Process GO term electron transport chain (ETC) to exclusively filter mitochondrial proteins.

Out of the identified 702 proteins, 43 belonged to ETC GO term and 86% of them showed higher levels (95% of these with a significant p-value) in the MB compared to the DC mitochondrial samples (Fig. 4B and Table S1). To further investigate non-mitochondrial organelle contaminations, the 702 identified proteins were also grouped in the following Cellular component GO terms: peroxisome, ER and microsomes. Microsomes are an artificial term, summarizing double membrane fragments mostly originating from ER during organelle isolations. Among all proteins, 50 peroxisomal, 143 ER and 64 microsomal proteins were found, respectively. Our data showed that 88% of peroxisomal, 82% of ER and 83% of microsomal proteins had a lower abundance in MB compared to DC groups and 94%, 83% and 67% of these peroxisomal, ER and microsomal proteins showed significantly lower levels in MB fractions, respectively (Fig. 4C–D and Table S1). However, a minority of non-mitochondrial proteins showed a higher level in MB fractions compared to DC samples. One should also note that a few proteins belonged to more than one GO term, while the localization of some proteins is not exclusively restricted to one organelle [25]. In conclusion, the isolation of liver mitochondria using automated tissue dissociation and MB mitochondrial enrichment has a clear benefit compared to the usual differential centrifugation method, since it yields isolated fractions which are not only better enriched in mitochondrial proteins but also contain less non-mitochondrial contaminants.

### Liver Mitochondria Isolated by Magnetic Beads have Higher Oxygen Consumption Rates Compared to those Isolated by Differential Centrifugation

**Figure 5:**
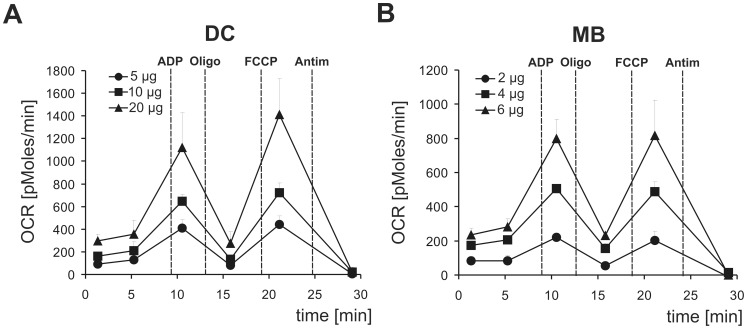
Liver mitochondria isolated by magnetic beads have higher oxygen consumption rates than mitochondria isolated by differential centrifugation. Oxygen consumption rates (OCRs) were determined using an XF24 Extracellular Flux Analyzer with the given amount of freshly isolated mitochondria (µg/well), which were purified by **A** DC or **B** MB methods. Briefly, succinate was used as a substrate to feed electrons to complex II and basal (state II) respiration was measured. State III respiration was determined in the presence of ADP. Oligomycin (Oligo) addition blocked ATP synthase and so evoked state IVo respiration. Maximum respiration (state uncoupled) was measured in the presence of the uncoupler FCCP. Finally, Antimycin A (Antim) was given to inhibit complex III. Data are represented as means of three independent experiments (5–8 replicates per groups) ± SD.

Since new, high-throughput oxygen consumption assays have emerged, that have important advantages compared to the traditional Clark electrode, we compared oxygen consumption of DC and MB liver mitochondria with the XF Extracellular Flux Analyzer. By using increasing amounts of mitochondria, we showed a good dependence of oxygen consumption rates (OCR) vs. protein input in both mitochondrial fractions (Fig. 5). Probably because MB mitochondria had a higher purity compared to DC extractions (Fig. 4), less material was required to reach a similar OCR range: 2–6 µg of MB mitochondria consumed as much oxygen as 5–20 µg of DC mitochondria. Both mitochondrial fractions had a good membrane integrity, as judged by the obtained RCR values (state uncoupled/state IVo: 5.3±0.2 for DC and 3.5±0.3 for MB mitochondria, respectively). In conclusion, these data indicate that automated tissue dissociation and magnetic beads isolation of liver mitochondria result in mitochondrial preparations with higher respiratory activity (normalized to protein mass) and higher purity (devoid of ER and peroxisomal contamination) compared to the conventional DC method.

## Discussion

In this study, we developed a new protocol for isolating functional mitochondria from different mouse tissues. Although mitochondrial isolation techniques are widely used, there is no consensus among them. The classical mitochondrial isolation protocols alternatively apply Dounce or Elvehjem homogenizers for tissue disruption [14], [15]. In principle, the pestle is usually moved into the homogenizing tube containing the tissue fractions either manually or using a motor drive, therefore the force and speed used is either difficult to control and standardize, as these depend greatly on the experience of the operator performing the homogenization, or varies from lab to lab. It has previously been demonstrated that RCR values of mitochondrial fractions directly correlate to the level of experience of the operators [26]. In addition, increasing the number of strokes usually leads to a higher mitochondrial yield, although the chance that concomitant mitochondrial membrane damage occurs is also higher [27]. Using the differential centrifugation method, non-mitochondrial organelles such as peroxisomes, ER and microsomes are usually co-purified [15]. Since these organelle contaminants can be present in a significant amount, especially in the case of liver tissue, they can lead to an overestimation of the total mitochondrial protein concentration.

With the aim of developing a reproducible protocol to specifically purify mitochondria from mouse tissue, we combined the anti-TOM22 magnetic beads (MB) isolation technique with an automated tissue dissociator. As the GentleMACS® Dissociator has fixed instrument settings for homogenization, tissue disruption is performed consistently and thus does not depend on the operator. In addition, this method is suitable for the automated homogenization of multiple samples simultaneously. We tested the homogenization steps for different tissues and the integrity of the mitochondrial membranes was assessed by transmission EM analysis, oxygen consumption measurements and determination of the respiratory control ratio (RCR). Transmission EM analysis revealed homogenous mitochondrial fractions with a good membrane integrity and purity isolated by anti-TOM22 magnetic beads from different mouse tissues. Our Western blot and Clark electrode assays confirmed the high purity and functionality of the mitochondria. Notably, mitochondrial isolated either from soft (liver and brain) or hard (skeletal muscle) tissues showed reliable functionality. In addition, we compared the purity and oxygen consumption capacity of liver mitochondria isolated with the usual differential centrifugation (DC) or magnetic beads (MB) methods. Our proteomics, Western blot and XF Extracellular Flux Analyzer analyses indicated that the MB liver mitochondrial fraction contained more mitochondrial and less non-mitochondrial (ER, microsomal and peroxisomal) contaminants. Moreover, most likely due to this increased purity, they also showed elevated oxygen consumption capacity compared to DC isolated mitochondria. On the other hand DC isolated liver mitochondria showed higher RCR, which could indicate that MB purified mitochondria are slightly more leaky for hydrogen ions but according to our experience the obtained 3.5 ratio still represents a good membrane integrity.

Since the traditional Clark electrode can only measure one sample simultaneously and mitochondrial measurements in this system need continuous manipulations, a new automated, high-throughput technique has been introduced [9]. With the XF Extracellular Flux Analyzer one can measure 24 (XF24) or 96 (XF96) samples in parallel. However, such measurements with high sample numbers require a reproducible technique for isolating pure mitochondria simultaneously from multiple tissue samples, which is less work intensive than the differential centrifugation protocol. Since mitochondria isolated from tissues by the combination of methods described here showed a reliable quality and quantity and these methods allow easy handling of numerous biological samples simultaneously, we propose that our approach will be valuable for many high-throughput downstream applications.

## Supporting Information

Table S1 **Identity of the proteins shown in** **Fig. 4B–D** **.** Liver mitochondrial fractions were isolated by the magnetic bead (MB) or differential centrifugation (DC) methods and proteins were identified by mass spectrometry. Protein ratios were calculated from protein abundances between MB and DC fractions and are represented as a linear ratio (average of MB/average of DC). When these ratios were less than 1 (DC average>MB average) the negative inverse ratio was taken and the values were transposed by the equation: −1/(MB/DC ratio). All ratios are also depicted in Fig. 4B–D and the identified protein names are shown in this table. Numbers indicate the order of the proteins in Fig. 4B–D from left to right. Statistical analyzes were made by Mann-Whitney U test and p values were corrected to multiple testing according to Benjamini&Hochberg. A false discovery rate (FDR) <10% was set as the significance level and significant proteins are denoted by stars, ns: no significance.
(DOC)

## References

[1] Vafai SB, Mootha VK (2012) Mitochondrial disorders as windows into an ancient organelle. Nature 491: 374–383. 23151580 10.1038/nature11707

[2] Zsurka G, Kunz WS (2010) Mitochondrial dysfunction in neurological disorders with epileptic phenotypes. J Bioenerg Biomembr 42: 443–448. 21069442 10.1007/s10863-010-9314-7

[3] Ekstrand MI, Terzioglu M, Galter D, Zhu S, Hofstetter C, et al (2007) Progressive parkinsonism in mice with respiratory-chain-deficient dopamine neurons. Proc Natl Acad Sci U S A 104: 1325–1330. 17227870 10.1073/pnas.0605208103 PMC1783140

[4] Park CB, Larsson NG (2011) Mitochondrial DNA mutations in disease and aging. J Cell Biol 193: 809–818. 21606204 10.1083/jcb.201010024 PMC3105550

[5] Szendroedi J, Schmid AI, Chmelik M, Toth C, Brehm A, et al (2007) Muscle mitochondrial ATP synthesis and glucose transport/phosphorylation in type 2 diabetes. PLoS Med 4: e154. 17472434 10.1371/journal.pmed.0040154 PMC1858707

[6] Szendroedi J, Chmelik M, Schmid AI, Nowotny P, Brehm A, et al (2009) Abnormal hepatic energy homeostasis in type 2 diabetes. Hepatology 50: 1079–1086. 19637187 10.1002/hep.23093

[7] Bonnard C, Durand A, Peyrol S, Chanseaume E, Chauvin MA, et al (2008) Mitochondrial dysfunction results from oxidative stress in the skeletal muscle of diet-induced insulin-resistant mice. J Clin Invest 118: 789–800. 18188455 10.1172/JCI32601 PMC2176186

[8] Franko A, von Kleist-Retzow JC, Bose M, Sanchez-Lasheras C, Brodesser S, et al (2012) Complete failure of insulin-transmitted signaling, but not obesity-induced insulin resistance, impairs respiratory chain function in muscle. J Mol Med (Berl) 90: 1145–1160. 22411022 10.1007/s00109-012-0887-y

[9] Horan MP, Pichaud N, Ballard JW (2012) Review: quantifying mitochondrial dysfunction in complex diseases of aging. J Gerontol A Biol Sci Med Sci 67: 1022–1035. 22459622 10.1093/gerona/glr263

[10] Rogers GW, Brand MD, Petrosyan S, Ashok D, Elorza AA, et al (2011) High throughput microplate respiratory measurements using minimal quantities of isolated mitochondria. PLoS One 6: e21746. 21799747 10.1371/journal.pone.0021746 PMC3143121

[11] Sims NR (1990) Rapid isolation of metabolically active mitochondria from rat brain and subregions using Percoll density gradient centrifugation. J Neurochem 55: 698–707. 2164576 10.1111/j.1471-4159.1990.tb04189.x

[12] Sims NR, Anderson MF (2008) Isolation of mitochondria from rat brain using Percoll density gradient centrifugation. Nat Protoc 3: 1228–1239. 18600228 10.1038/nprot.2008.105

[13] Hartwig S, Feckler C, Lehr S, Wallbrecht K, Wolgast H, et al (2009) A critical comparison between two classical and a kit-based method for mitochondria isolation. Proteomics 9: 3209–3214. 19415664 10.1002/pmic.200800344

[14] Frezza C, Cipolat S, Scorrano L (2007) Organelle isolation: functional mitochondria from mouse liver, muscle and cultured fibroblasts. Nat Protoc 2: 287–295. 17406588 10.1038/nprot.2006.478

[15] Fernandez-Vizarra E, Ferrin G, Perez-Martos A, Fernandez-Silva P, Zeviani M, et al (2010) Isolation of mitochondria for biogenetical studies: An update. Mitochondrion 10: 253–262. 20034597 10.1016/j.mito.2009.12.148

[16] Gross VS, Greenberg HK, Baranov SV, Carlson GM, Stavrovskaya IG, et al (2011) Isolation of functional mitochondria from rat kidney and skeletal muscle without manual homogenization. Anal Biochem 418: 213–223. 21820998 10.1016/j.ab.2011.07.017 PMC3172370

[17] Hornig-Do HT, Gunther G, Bust M, Lehnartz P, Bosio A, et al (2009) Isolation of functional pure mitochondria by superparamagnetic microbeads. Anal Biochem 389: 1–5. 19285029 10.1016/j.ab.2009.02.040

[18] Rustin P, Chretien D, Bourgeron T, Gerard B, Rotig A, et al (1994) Biochemical and molecular investigations in respiratory chain deficiencies. Clin Chim Acta 228: 35–51. 7955428 10.1016/0009-8981(94)90055-8

[19] Merl J, Ueffing M, Hauck SM, von Toerne C (2012) Direct comparison of MS-based label-free and SILAC quantitative proteome profiling strategies in primary retinal Muller cells. Proteomics 12: 1902–1911. 22623344 10.1002/pmic.201100549

[20] von Toerne C, Kahle M, Schafer A, Ispiryan R, Blindert M, et al (2013) Apoe, Mbl2, and Psp plasma protein levels correlate with diabetic phenotype in NZO mice–an optimized rapid workflow for SRM-based quantification. J Proteome Res 12: 1331–1343. 23350727 10.1021/pr3009836

[21] Hauck SM, Dietter J, Kramer RL, Hofmaier F, Zipplies JK, et al (2010) Deciphering membrane-associated molecular processes in target tissue of autoimmune uveitis by label-free quantitative mass spectrometry. Mol Cell Proteomics 9: 2292–2305. 20601722 10.1074/mcp.M110.001073 PMC2953921

[22] Brosch M, Yu L, Hubbard T, Choudhary J (2009) Accurate and sensitive peptide identification with Mascot Percolator. J Proteome Res 8: 3176–3181. 19338334 10.1021/pr800982s PMC2734080

[23] Rainer J, Sanchez-Cabo F, Stocker G, Sturn A, Trajanoski Z (2006) CARMAweb: comprehensive R- and bioconductor-based web service for microarray data analysis. Nucleic Acids Res 34: W498–503. 16845058 10.1093/nar/gkl038 PMC1538903

[24] Laurindo FR, Pescatore LA, Fernandes Dde C (2012) Protein disulfide isomerase in redox cell signaling and homeostasis. Free Radic Biol Med 52: 1954–1969. 22401853 10.1016/j.freeradbiomed.2012.02.037

[25] Pagliarini DJ, Calvo SE, Chang B, Sheth SA, Vafai SB, et al (2008) A mitochondrial protein compendium elucidates complex I disease biology. Cell 134: 112–123. 18614015 10.1016/j.cell.2008.06.016 PMC2778844

[26] Rasmussen HN, Andersen AJ, Rasmussen UF (1997) Optimization of preparation of mitochondria from 25–100 mg skeletal muscle. Anal Biochem 252: 153–159. 9324953 10.1006/abio.1997.2304

[27] Kristian T, Hopkins IB, McKenna MC, Fiskum G (2006) Isolation of mitochondria with high respiratory control from primary cultures of neurons and astrocytes using nitrogen cavitation. J Neurosci Methods 152: 136–143. 16253339 10.1016/j.jneumeth.2005.08.018 PMC2572758

