# Supplementary material for: Efficient Isolation of Pure and Functional Mitochondria from Mouse Tissues Using Automated Tissue Disruption and Enrichment with Anti-TOM22 Magnetic Beads
Source: PLoS One. 2013 Dec 12;8(12):e82392. doi: 10.1371/journal.pone.0082392 (PMC3861405; doi:10.1371/journal.pone.0082392)
Supplement: Table S1 — Identity of the proteins shown in Fig. 4B–D . Liver mitochondrial fractions were isolated by the magnetic bead (MB) or differential centrifugation (DC) methods and proteins were identified by mass spectrometry. Protein ratios were calculated from protein abundances between MB and DC fractions and are represented as a linear ratio (average of MB/average of DC). When these ratios were less than 1 (DC average>MB average) the negative inverse ratio was taken and the values were transposed by the equation: −1/(MB/DC ratio). All ratios are also depicted in Fig. 4B–D and the identified protein names are shown in this table. Numbers indicate the order of the proteins in Fig. 4B–D from left to right. Statistical analyzes were made by Mann-Whitney U test and p values were corrected to multiple testing according to Benjamini&Hochberg. A false discovery rate (FDR) <10% was set as the significance level and significant proteins are denoted by stars, ns: no significance. (DOC) [file pone.0082392.s001.doc]

| **GO term: ETC** | **Number** | **Accession** | **Peptide count** | **Peptides used for quantitation** | **Gene ID** | **Linear ratio** | **Statistic** |
| --- | --- | --- | --- | --- | --- | --- | --- |
|  | **1** | ENSMUSP00000025549;ENSMUSP00000124412 | 7 | 7 | Cyb5 | **-1,74** | ***** |
|  | **2** | ENSMUSP00000034400 | 4 | 4 | Cyb5b | **-1,21** | ns |
|  | **3** | ENSMUSP00000025567 | 4 | 4 | Fads2 | **-1,15** | ns |
|  | **4** | ENSMUSP00000026222;ENSMUSP00000128192 | 4 | 4 | Ndufb8 | **2,03** | ***** |
|  | **5** | ENSMUSP00000040591 | 11 | 10 | Aldh5a1 | **1,58** | ns |
|  | **6** | ENSMUSP00000023210 | 11 | 11 | Cyc1 | **2,32** | ***** |
|  | **7** | ENSMUSP00000106481 | 18 | 18 | Dld | **2,08** | ***** |
|  | **8** | ENSMUSP00000034866 | 21 | 19 | Etfa | **2,44** | ***** |
|  | **9** | ENSMUSP00000004729 | 13 | 12 | Etfb | **1,66** | ***** |
|  | **10** | ENSMUSP00000029386 | 16 | 16 | Etfdh | **1,79** | ***** |
|  | **11** | ENSMUSP00000034552 | 2 | 2 | Fdx1 | **-1,24** | ns |
|  | **12** | ENSMUSP00000039487 | 2 | 2 | Frrs1 | **-4,39** | ***** |
|  | **13** | ENSMUSP00000027478 | 10 | 10 | Ndufa10 | **2,59** | ***** |
|  | **14** | ENSMUSP00000020209 | 3 | 3 | Ndufa12 | **1,93** | ***** |
|  | **15** | ENSMUSP00000105796 | 3 | 3 | Ndufa13 | **1,55** | ***** |
|  | **16** | ENSMUSP00000014438 | 2 | 2 | Ndufa2 | **2,60** | ***** |
|  | **17** | ENSMUSP00000023851;ENSMUSP00000112971 | 3 | 3 | Ndufa5 | **2,37** | ***** |
|  | **18** | ENSMUSP00000023085 | 2 | 2 | Ndufa6 | **5,58** | ***** |
|  | **19** | ENSMUSP00000065352 | 6 | 6 | Ndufa8 | **1,99** | ***** |
|  | **20** | ENSMUSP00000085523 | 13 | 13 | Ndufa9 | **1,88** | ***** |
|  | **21** | ENSMUSP00000043543;ENSMUSP00000120091 | 4 | 4 | Ndufb10 | **1,82** | ***** |
|  | **22** | ENSMUSP00000113602;ENSMUSP00000113169 | 3 | 3 | Ndufb5 | **2,01** | ***** |
|  | **23** | ENSMUSP00000092746;ENSMUSP00000103743 | 3 | 3 | Ndufb6 | **1,70** | ***** |
|  | **24** | ENSMUSP00000022980 | 7 | 7 | Ndufb9 | **1,88** | ***** |
|  | **25** | ENSMUSP00000027111 | 22 | 22 | Ndufs1 | **2,14** | ***** |
|  | **26** | ENSMUSP00000013737;ENSMUSP00000121397 | 12 | 12 | Ndufs2 | **-1,09** | ns |
|  | **27** | ENSMUSP00000005647 | 10 | 10 | Ndufs3 | **1,92** | ***** |
|  | **28** | ENSMUSP00000022286 | 2 | 2 | Ndufs4 | **2,31** | ***** |
|  | **29** | ENSMUSP00000020361 | 3 | 3 | Ndufs7 | **1,49** | ***** |
|  | **30** | ENSMUSP00000074600 | 4 | 4 | Ndufs8 | **1,69** | ***** |
|  | **31** | ENSMUSP00000042967;ENSMUSP00000115653 | 18 | 17 | Ndufv1 | **2,03** | ***** |
|  | **32** | ENSMUSP00000121557;ENSMUSP00000024909 | 5 | 5 | Ndufv2 | **2,40** | ***** |
|  | **33** | ENSMUSP00000022062 | 35 | 33 | Sdha | **2,51** | ***** |
|  | **34** | ENSMUSP00000010007 | 9 | 9 | Sdhb | **2,06** | ***** |
|  | **35** | ENSMUSP00000106968;ENSMUSP00000080273 | 3 | 3 | Sdhc | **2,55** | ***** |
|  | **36** | ENSMUSP00000122103 | 10 | 6 | Slc25a12 | **2,98** | ***** |
|  | **37** | ENSMUSP00000015256 | 29 | 25 | Slc25a13 | **2,19** | ***** |
|  | **38** | ENSMUSP00000007012 | 9 | 9 | Sod2 | **1,92** | ***** |
|  | **39** | ENSMUSP00000054856 | 2 | 2 | Uqcr10 | **1,32** | ns |
|  | **40** | ENSMUSP00000026743 | 22 | 21 | Uqcrc1 | **2,71** | ***** |
|  | **41** | ENSMUSP00000033176;ENSMUSP00000041732 | 24 | 22 | Uqcrc2 | **3,00** | ***** |
|  | **42** | ENSMUSP00000045284 | 8 | 8 | Uqcrfs1 | **2,51** | ***** |
|  | **43** | ENSMUSP00000053145 | 4 | 4 | Uqcrq | **1,83** | ***** |
|  |  |  |  |  |  |  |  |
|  |  |  |  |  |  |  |  |
| **GO term: peroxisome** | **Number** | **Accession** | **Peptide count** | **Peptides used for quantitation** | **Gene ID** | **Linear ratio** | **Statistic** |
|  | **1** | ENSMUSP00000026554 | 3 | 3 | 1190003J15Rik | **-3,87** | ns |
|  | **2** | ENSMUSP00000029770 | 30 | 30 | Abcd3 | **-1,67** | ns |
|  | **3** | ENSMUSP00000042351;ENSMUSP00000134926 | 30 | 6 | Acaa1a | **-2,73** | ***** |
|  | **4** | ENSMUSP00000010795 | 33 | 11 | Acaa1b | **-2,07** | ***** |
|  | **5** | ENSMUSP00000043424;ENSMUSP00000075476 | 19 | 18 | Acad11 | **-2,71** | ***** |
|  | **6** | ENSMUSP00000028121;ENSMUSP00000117325 | 4 | 4 | Acbd5 | **1,05** | ***** |
|  | **7** | ENSMUSP00000092702;ENSMUSP00000080256 | 6 | 6 | Acnat1 | **-3,58** | ***** |
|  | **8** | ENSMUSP00000017451 | 3 | 2 | Acot8 | **-4,38** | ***** |
|  | **9** | ENSMUSP00000063325;ENSMUSP00000122185 | 51 | 2 | Acox1 | **-1,87** | ***** |
|  | **10** | ENSMUSP00000022271;ENSMUSP00000130543 | 23 | 23 | Acox2 | **-2,72** | ***** |
|  | **11** | ENSMUSP00000034046;ENSMUSP00000114473 | 37 | 31 | Acsl1 | **-1,22** | ns |
|  | **12** | ENSMUSP00000027491 | 11 | 11 | Agxt | **-1,24** | ns |
|  | **13** | ENSMUSP00000066915 | 12 | 11 | Amacr | **-2,04** | ***** |
|  | **14** | ENSMUSP00000041983 | 14 | 14 | Baat | **-2,39** | ***** |
|  | **15** | ENSMUSP00000028610;ENSMUSP00000106798 | 62 | 62 | Cat | **-5,56** | ***** |
|  | **16** | ENSMUSP00000003720 | 12 | 12 | Crot | **-3,22** | ***** |
|  | **17** | ENSMUSP00000045621 | 11 | 10 | Decr2 | **-4,05** | ***** |
|  | **18** | ENSMUSP00000022821 | 9 | 9 | Dhrs4 | **-1,96** | ***** |
|  | **19** | ENSMUSP00000044924 | 2 | 2 | Dhrs7b | **-2,64** | ***** |
|  | **20** | ENSMUSP00000066092;ENSMUSP00000116992 | 12 | 10 | Ech1 | **1,02** | ns |
|  | **21** | ENSMUSP00000021854;ENSMUSP00000021853 | 8 | 7 | Eci2 | **-1,25** | ns |
|  | **22** | ENSMUSP00000023559 | 46 | 44 | Ehhadh | **-3,22** | ***** |
|  | **23** | ENSMUSP00000069209 | 38 | 36 | Ephx2 | **-2,33** | ***** |
|  | **24** | ENSMUSP00000064655 | 10 | 10 | Fabp1 | **-2,61** | ns |
|  | **25** | ENSMUSP00000022437;ENSMUSP00000132913 | 19 | 18 | Hacl1 | **-4,15** | ***** |
|  | **26** | ENSMUSP00000028704 | 16 | 16 | Hao1 | **-3,75** | ***** |
|  | **27** | ENSMUSP00000030432 | 10 | 10 | Hmgcl | **1,26** | ns |
|  | **28** | ENSMUSP00000025385 | 29 | 27 | Hsd17b4 | **-3,14** | ***** |
|  | **29** | ENSMUSP00000030078 | 2 | 2 | Hsdl2 | **1,10** | ns |
|  | **30** | ENSMUSP00000095316;ENSMUSP00000117853 | 12 | 11 | Idh1 | **-2,26** | ***** |
|  | **31** | ENSMUSP00000034141;ENSMUSP00000113381 | 16 | 15 | Lonp2 | **-3,52** | ***** |
|  | **32** | ENSMUSP00000008684;ENSMUSP00000112646 | 12 | 12 | Mgst1 | **1,30** | ns |
|  | **33** | ENSMUSP00000095970 | 6 | 3 | Mlycd | **-1,23** | ***** |
|  | **34** | ENSMUSP00000025065;ENSMUSP00000133678 | 5 | 3 | Nudt12 | **-2,19** | ***** |
|  | **35** | ENSMUSP00000047778 | 6 | 6 | Nudt19 | **-2,01** | ***** |
|  | **36** | ENSMUSP00000073213;ENSMUSP00000034382 | 8 | 8 | Nudt7 | **-4,31** | ***** |
|  | **37** | ENSMUSP00000027381;ENSMUSP00000120890 | 8 | 8 | Pecr | **-1,90** | ***** |
|  | **38** | ENSMUSP00000099506 | 3 | 3 | Pex14 | **-2,39** | ***** |
|  | **39** | ENSMUSP00000002840 | 2 | 2 | Pex6 | **-1,63** | ns |
|  | **40** | ENSMUSP00000027975 | 9 | 9 | Phyh | **-3,54** | ***** |
|  | **41** | ENSMUSP00000017597 | 16 | 16 | Pipox | **-3,01** | ***** |
|  | **42** | ENSMUSP00000102078;ENSMUSP00000030454 | 7 | 5 | Prdx1 | **-1,44** | ***** |
|  | **43** | ENSMUSP00000025904;ENSMUSP00000134521 | 9 | 9 | Prdx5 | **1,31** | ***** |
|  | **44** | ENSMUSP00000031472 | 8 | 8 | Pxmp2 | **-4,24** | ***** |
|  | **45** | ENSMUSP00000030340;ENSMUSP00000102312 | 45 | 45 | Scp2 | **-3,12** | ***** |
|  | **46** | ENSMUSP00000057595;ENSMUSP00000117145 | 22 | 22 | Slc27a2 | **-1,24** | ns |
|  | **47** | ENSMUSP00000023707 | 5 | 5 | Sod1 | **-10,74** | ***** |
|  | **48** | ENSMUSP00000020284 | 2 | 2 | Tysnd1 | **-12,01** | ***** |
|  | **49** | ENSMUSP00000029837;ENSMUSP00000113649 | 39 | 39 | Uox | **-4,85** | ***** |
|  | **50** | ENSMUSP00000052544 | 5 | 5 | Zadh2 | **-1,06** | ns |
|  |  |  |  |  |  |  |  |
|  |  |  |  |  |  |  |  |
| **GO term: microsome** | **Number** | **Accession** | **Peptide count** | **Peptides used for quantitation** | **Gene ID** | **Linear ratio** | **Statistic** |
|  | **1** | ENSMUSP00000029325 | 20 | 19 | Aadac | **-1,80** | ***** |
|  | **2** | ENSMUSP00000041104 | 19 | 18 | Aifm1 | **1,48** | ***** |
|  | **3** | ENSMUSP00000067767;ENSMUSP00000006792 | 20 | 20 | Aldh3a2 | **-1,73** | ***** |
|  | **4** | ENSMUSP00000029987 | 7 | 6 | Aldob | **-2,63** | ***** |
|  | **5** | ENSMUSP00000036044;ENSMUSP00000035761 | 5 | 3 | Apob | **-1,36** | ns |
|  | **6** | ENSMUSP00000039657;ENSMUSP00000005692 | 23 | 22 | Atp1a1 | **-1,82** | ***** |
|  | **7** | ENSMUSP00000031423;ENSMUSP00000032974 | 6 | 6 | Atp2a2 | **-1,36** | ***** |
|  | **8** | ENSMUSP00000003912 | 18 | 18 | Calr | **-3,41** | ***** |
|  | **9** | ENSMUSP00000002043;ENSMUSP00000102478 | 5 | 5 | Ccdc47 | **-1,54** | ***** |
|  | **10** | ENSMUSP00000034346;ENSMUSP00000076988 | 28 | 20 | Ces2a | **-3,16** | ***** |
|  | **11** | ENSMUSP00000025835 | 9 | 9 | Cpt1a | **1,27** | ns |
|  | **12** | ENSMUSP00000025549;ENSMUSP00000124412 | 7 | 7 | Cyb5 | **-1,74** | ***** |
|  | **13** | ENSMUSP00000034400 | 4 | 4 | Cyb5b | **-1,21** | ns |
|  | **14** | ENSMUSP00000034860;ENSMUSP00000034865 | 26 | 24 | Cyp1a2 | **-1,28** | ns |
|  | **15** | ENSMUSP00000074990;ENSMUSP00000128030 | 21 | 16 | Cyp2a12 | **-1,48** | ns |
|  | **16** | ENSMUSP00000003137;ENSMUSP00000135839 | 25 | 12 | Cyp2c29 | **-1,30** | ns |
|  | **17** | ENSMUSP00000026211 | 15 | 13 | Cyp2c44 | **1,03** | ns |
|  | **18** | ENSMUSP00000079065;ENSMUSP00000068039 | 20 | 4 | Cyp2c50 | **1,39** | ***** |
|  | **19** | ENSMUSP00000023083 | 13 | 6 | Cyp2d22 | **-1,56** | ***** |
|  | **20** | ENSMUSP00000006094;ENSMUSP00000060524 | 24 | 16 | Cyp2d26 | **-2,34** | ***** |
|  | **21** | ENSMUSP00000086530 | 19 | 7 | Cyp2d9 | **-1,64** | ***** |
|  | **22** | ENSMUSP00000026552 | 28 | 25 | Cyp2e1 | **-1,55** | ns |
|  | **23** | ENSMUSP00000003100 | 33 | 31 | Cyp2f2 | **-1,10** | ns |
|  | **24** | ENSMUSP00000037665;ENSMUSP00000031633 | 31 | 14 | Cyp3a11 | **-1,01** | ns |
|  | **25** | ENSMUSP00000081370 | 13 | 2 | Cyp4a12a | **-1,37** | ns |
|  | **26** | ENSMUSP00000092487;ENSMUSP00000030480 | 13 | 2 | Cyp4a12b | **-1,50** | ***** |
|  | **27** | ENSMUSP00000030487 | 5 | 2 | Cyp4a14 | **-1,80** | ***** |
|  | **28** | ENSMUSP00000001507 | 4 | 4 | Cyp51 | **-1,48** | ns |
|  | **29** | ENSMUSP00000037487 | 10 | 10 | Cyp7b1 | **1,13** | ns |
|  | **30** | ENSMUSP00000030538 | 12 | 11 | Ddost | **-1,40** | ns |
|  | **31** | ENSMUSP00000060346 | 8 | 7 | Dlst | **1,83** | ***** |
|  | **32** | ENSMUSP00000021793;ENSMUSP00000114112 | 2 | 2 | Elovl2 | **1,18** | ***** |
|  | **33** | ENSMUSP00000047551;ENSMUSP00000106697 | 26 | 26 | Ephx1 | **-2,34** | ns |
|  | **34** | ENSMUSP00000133166 | 7 | 7 | Erap1 | **-4,22** | ***** |
|  | **35** | ENSMUSP00000037259;ENSMUSP00000107143 | 15 | 15 | Fmo1 | **-1,72** | ***** |
|  | **36** | ENSMUSP00000083375;ENSMUSP00000114377 | 2 | 2 | Glul | **-1,66** | ***** |
|  | **37** | ENSMUSP00000026613;ENSMUSP00000106939 | 10 | 9 | Gusb | **-6,49** | ***** |
|  | **38** | ENSMUSP00000030830;ENSMUSP00000115647 | 13 | 12 | H6pd | **-1,70** | ***** |
|  | **39** | ENSMUSP00000016338;ENSMUSP00000124693 | 13 | 13 | Hsd11b1 | **-2,02** | ***** |
|  | **40** | ENSMUSP00000034304 | 10 | 10 | Hsd17b2 | **1,19** | ns |
|  | **41** | ENSMUSP00000036245;ENSMUSP00000114536 | 3 | 3 | Hsd3b7 | **-1,28** | ns |
|  | **42** | ENSMUSP00000020238;ENSMUSP00000122710 | 33 | 27 | Hsp90b1 | **-2,61** | ***** |
|  | **43** | ENSMUSP00000019896 | 3 | 3 | Iyd | **-1,51** | ns |
|  | **44** | ENSMUSP00000040140;ENSMUSP00000035610 | 11 | 10 | Lman1 | **-1,97** | ***** |
|  | **45** | ENSMUSP00000046856 | 3 | 3 | Lss | **-1,35** | ***** |
|  | **46** | ENSMUSP00000008684;ENSMUSP00000112646 | 12 | 12 | Mgst1 | **1,30** | ns |
|  | **47** | ENSMUSP00000026122;ENSMUSP00000128796 | 34 | 32 | P4hb | **-2,98** | ***** |
|  | **48** | ENSMUSP00000002663;ENSMUSP00000135195 | 8 | 8 | Pon1 | **-2,45** | ***** |
|  | **49** | ENSMUSP00000062670 | 2 | 2 | Pon2 | **-1,33** | ns |
|  | **50** | ENSMUSP00000031773;ENSMUSP00000135603 | 8 | 8 | Pon3 | **-2,32** | ns |
|  | **51** | ENSMUSP00000005651;ENSMUSP00000112924 | 32 | 31 | Por | **-2,15** | ***** |
|  | **52** | ENSMUSP00000032143 | 18 | 17 | Rpn1 | **-1,72** | ***** |
|  | **53** | ENSMUSP00000112081;ENSMUSP00000029171 | 15 | 15 | Rpn2 | **-1,51** | ***** |
|  | **54** | ENSMUSP00000032168 | 2 | 2 | Sec61a1 | **1,96** | ***** |
|  | **55** | ENSMUSP00000057595;ENSMUSP00000117145 | 22 | 22 | Slc27a2 | **-1,24** | ns |
|  | **56** | ENSMUSP00000032539;ENSMUSP00000112495 | 23 | 22 | Slc27a5 | **-1,22** | ns |
|  | **57** | ENSMUSP00000037583 | 3 | 3 | Tmed10 | **-1,03** | ns |
|  | **58** | ENSMUSP00000072803;ENSMUSP00000037258 | 27 | 14 | Ugt1a1 | **-1,99** | ***** |
|  | **59** | ENSMUSP00000014263 | 20 | 3 | Ugt1a6a | **-1,49** | ns |
|  | **60** | ENSMUSP00000073444 | 17 | 5 | Ugt1a9 | **-1,74** | ***** |
|  | **61** | ENSMUSP00000031183 | 27 | 22 | Ugt2b1 | **-1,48** | ns |
|  | **62** | ENSMUSP00000031186 | 7 | 3 | Ugt2b35 | **-1,38** | ns |
|  | **63** | ENSMUSP00000030164;ENSMUSP00000026486 | 5 | 5 | Vcp | **-2,07** | ns |
|  | **64** | ENSMUSP00000009036;ENSMUSP00000136273 | 7 | 5 | Vdac3 | **1,47** | ***** |
|  |  |  |  |  |  |  |  |
|  |  |  |  |  |  |  |  |
| **GO term: ER** | **Number** | **Accession** | **Peptide count** | **Peptides used for quantitation** | **Gene ID** | **Linear ratio** | **Statistic** |
|  | **1** | ENSMUSP00000029325 | 20 | 19 | Aadac | **-1,80** | ***** |
|  | **2** | ENSMUSP00000034046;ENSMUSP00000114473 | 37 | 31 | Acsl1 | **-1,22** | ns |
|  | **3** | ENSMUSP00000046585 | 26 | 24 | Acsl5 | **-1,23** | ns |
|  | **4** | ENSMUSP00000044012;ENSMUSP00000123877 | 46 | 45 | Agl | **-6,95** | ***** |
|  | **5** | ENSMUSP00000067767;ENSMUSP00000006792 | 20 | 20 | Aldh3a2 | **-1,73** | ***** |
|  | **6** | ENSMUSP00000029987 | 7 | 6 | Aldob | **-2,63** | ***** |
|  | **7** | ENSMUSP00000036044;ENSMUSP00000035761 | 5 | 3 | Apob | **-1,36** | ns |
|  | **8** | ENSMUSP00000088964 | 3 | 3 | Arsb | **-2,00** | ***** |
|  | **9** | ENSMUSP00000123799;ENSMUSP00000123861 | 2 | 2 | Asl | **-2,59** | ***** |
|  | **10** | ENSMUSP00000077273;ENSMUSP00000049018 | 3 | 3 | Asph | **-1,94** | ***** |
|  | **11** | ENSMUSP00000099904;ENSMUSP00000136734 | 25 | 25 | Ass1 | **3,06** | ***** |
|  | **12** | ENSMUSP00000025668 | 4 | 4 | Atl3 | **-1,20** | ns |
|  | **13** | ENSMUSP00000033480 | 8 | 8 | Atp11c | **1,02** | ns |
|  | **14** | ENSMUSP00000031423;ENSMUSP00000032974 | 6 | 6 | Atp2a2 | **-1,36** | ***** |
|  | **15** | ENSMUSP00000002091 | 2 | 2 | Bcap31 | **-5,09** | ***** |
|  | **16** | ENSMUSP00000003912 | 18 | 18 | Calr | **-3,41** | ***** |
|  | **17** | ENSMUSP00000020637 | 12 | 12 | Canx | **-2,34** | ***** |
|  | **18** | ENSMUSP00000028610;ENSMUSP00000106798 | 62 | 62 | Cat | **-5,56** | ***** |
|  | **19** | ENSMUSP00000002043;ENSMUSP00000102478 | 5 | 5 | Ccdc47 | **-1,54** | ***** |
|  | **20** | ENSMUSP00000034189;ENSMUSP00000123146 | 7 | 3 | Ces1c | **-2,31** | ***** |
|  | **21** | ENSMUSP00000034172 | 32 | 27 | Ces1d | **-2,53** | ***** |
|  | **22** | ENSMUSP00000034173 | 11 | 6 | Ces1e | **-2,54** | ***** |
|  | **23** | ENSMUSP00000037555 | 13 | 10 | Ces1g | **-2,91** | ***** |
|  | **24** | ENSMUSP00000090910 | 28 | 17 | Ces3a | **-2,43** | ***** |
|  | **25** | ENSMUSP00000090909;ENSMUSP00000074004 | 17 | 8 | Ces3b | **-3,69** | ***** |
|  | **26** | ENSMUSP00000044587;ENSMUSP00000032073 | 5 | 4 | Cml2 | **-2,48** | ns |
|  | **27** | ENSMUSP00000026446 | 2 | 2 | Cnpy2 | **-1,84** | ns |
|  | **28** | ENSMUSP00000025835 | 9 | 9 | Cpt1a | **1,27** | ns |
|  | **29** | ENSMUSP00000032779;ENSMUSP00000119503 | 4 | 4 | Ctsc | **-5,26** | ***** |
|  | **30** | ENSMUSP00000016400 | 5 | 5 | Ctsz | **-13,51** | ***** |
|  | **31** | ENSMUSP00000025549;ENSMUSP00000124412 | 7 | 7 | Cyb5 | **-1,74** | ***** |
|  | **32** | ENSMUSP00000018186;ENSMUSP00000124062 | 22 | 22 | Cyb5r3 | **-1,57** | ***** |
|  | **33** | ENSMUSP00000034860;ENSMUSP00000034865 | 26 | 24 | Cyp1a2 | **-1,28** | ns |
|  | **34** | ENSMUSP00000074990;ENSMUSP00000128030 | 21 | 16 | Cyp2a12 | **-1,48** | ns |
|  | **35** | ENSMUSP00000003137;ENSMUSP00000135839 | 25 | 12 | Cyp2c29 | **-1,30** | ns |
|  | **36** | ENSMUSP00000045362;ENSMUSP00000084487 | 15 | 4 | Cyp2c37 | **1,06** | ns |
|  | **37** | ENSMUSP00000079065;ENSMUSP00000068039 | 20 | 4 | Cyp2c50 | **1,39** | ***** |
|  | **38** | ENSMUSP00000048284 | 19 | 7 | Cyp2c54 | **-1,40** | ns |
|  | **39** | ENSMUSP00000060584;ENSMUSP00000128691 | 14 | 12 | Cyp2c70 | **-1,40** | ***** |
|  | **40** | ENSMUSP00000072555 | 23 | 10 | Cyp2d10 | **-1,54** | ns |
|  | **41** | ENSMUSP00000006094;ENSMUSP00000060524 | 24 | 16 | Cyp2d26 | **-2,34** | ***** |
|  | **42** | ENSMUSP00000086530 | 19 | 7 | Cyp2d9 | **-1,64** | ***** |
|  | **43** | ENSMUSP00000026552 | 28 | 25 | Cyp2e1 | **-1,55** | ns |
|  | **44** | ENSMUSP00000003100 | 33 | 31 | Cyp2f2 | **-1,10** | ns |
|  | **45** | ENSMUSP00000030299;ENSMUSP00000030305 | 13 | 11 | Cyp2j5 | **1,29** | ns |
|  | **46** | ENSMUSP00000037665;ENSMUSP00000031633 | 31 | 14 | Cyp3a11 | **-1,01** | ns |
|  | **47** | ENSMUSP00000031741 | 6 | 4 | Cyp3a13 | **-1,04** | ns |
|  | **48** | ENSMUSP00000065585;ENSMUSP00000049494 | 2 | 2 | Cyp3a25 | **-1,13** | ns |
|  | **49** | ENSMUSP00000081370 | 13 | 2 | Cyp4a12a | **-1,37** | ns |
|  | **50** | ENSMUSP00000030487 | 5 | 2 | Cyp4a14 | **-1,80** | ***** |
|  | **51** | ENSMUSP00000092966;ENSMUSP00000026907 | 9 | 8 | Cyp4v3 | **-1,47** | ns |
|  | **52** | ENSMUSP00000001507 | 4 | 4 | Cyp51 | **-1,48** | ns |
|  | **53** | ENSMUSP00000037487 | 10 | 10 | Cyp7b1 | **1,13** | ns |
|  | **54** | ENSMUSP00000052989 | 3 | 3 | Cyp8b1 | **-1,57** | ***** |
|  | **55** | ENSMUSP00000030538 | 12 | 11 | Ddost | **-1,40** | ns |
|  | **56** | ENSMUSP00000002403 | 8 | 8 | Dhrs1 | **1,95** | ***** |
|  | **57** | ENSMUSP00000004574 | 4 | 4 | Dnajb11 | **1,39** | ***** |
|  | **58** | ENSMUSP00000022734 | 9 | 9 | Dnajc3 | **-2,65** | ***** |
|  | **59** | ENSMUSP00000044050 | 3 | 3 | Dpp4 | **-1,76** | ***** |
|  | **60** | ENSMUSP00000020329;ENSMUSP00000099948 | 10 | 9 | Egfr | **-2,32** | ***** |
|  | **61** | ENSMUSP00000021793;ENSMUSP00000114112 | 2 | 2 | Elovl2 | **1,18** | ***** |
|  | **62** | ENSMUSP00000021662 | 6 | 5 | Entpd5 | **-2,70** | ***** |
|  | **63** | ENSMUSP00000047551;ENSMUSP00000106697 | 26 | 26 | Ephx1 | **-2,34** | ns |
|  | **64** | ENSMUSP00000133166 | 7 | 7 | Erap1 | **-4,22** | ***** |
|  | **65** | ENSMUSP00000117347;ENSMUSP00000059275 | 2 | 2 | Erp29 | **-2,99** | ***** |
|  | **66** | ENSMUSP00000030028;ENSMUSP00000021091 | 9 | 9 | Erp44 | **-1,85** | ***** |
|  | **67** | ENSMUSP00000041543 | 11 | 10 | Faah | **-1,32** | ***** |
|  | **68** | ENSMUSP00000025567 | 4 | 4 | Fads2 | **-1,15** | ns |
|  | **69** | ENSMUSP00000066839 | 2 | 2 | Fkbp2 | **-4,27** | ***** |
|  | **70** | ENSMUSP00000037259;ENSMUSP00000107143 | 15 | 15 | Fmo1 | **-1,72** | ***** |
|  | **71** | ENSMUSP00000029729 | 24 | 23 | Fmo5 | **1,06** | ns |
|  | **72** | ENSMUSP00000093965 | 27 | 27 | Ganab | **-2,76** | ***** |
|  | **73** | ENSMUSP00000070109 | 4 | 4 | Ggcx | **-1,53** | ***** |
|  | **74** | ENSMUSP00000083375;ENSMUSP00000114377 | 2 | 2 | Glul | **-1,66** | ***** |
|  | **75** | ENSMUSP00000060912 | 17 | 17 | Gulo | **-1,54** | ***** |
|  | **76** | ENSMUSP00000026613;ENSMUSP00000106939 | 10 | 9 | Gusb | **-6,49** | ***** |
|  | **77** | ENSMUSP00000030830;ENSMUSP00000115647 | 13 | 12 | H6pd | **-1,70** | ***** |
|  | **78** | ENSMUSP00000031398;ENSMUSP00000121922 | 3 | 3 | Hpd | **-2,51** | ***** |
|  | **79** | ENSMUSP00000016338;ENSMUSP00000124693 | 13 | 13 | Hsd11b1 | **-2,02** | ***** |
|  | **80** | ENSMUSP00000026289;ENSMUSP00000108236 | 19 | 17 | Hsd17b10 | **2,38** | ***** |
|  | **81** | ENSMUSP00000028619 | 6 | 5 | Hsd17b12 | **1,79** | ***** |
|  | **82** | ENSMUSP00000088246;ENSMUSP00000029463 | 7 | 4 | Hsd3b3 | **1,36** | ***** |
|  | **83** | ENSMUSP00000041442;ENSMUSP00000120119 | 14 | 6 | Hsd3b5 | **1,99** | ***** |
|  | **84** | ENSMUSP00000036245;ENSMUSP00000114536 | 3 | 3 | Hsd3b7 | **-1,28** | ns |
|  | **85** | ENSMUSP00000020238;ENSMUSP00000122710 | 33 | 27 | Hsp90b1 | **-2,61** | ***** |
|  | **86** | ENSMUSP00000028222 | 38 | 34 | Hspa5 | **-3,22** | ***** |
|  | **87** | ENSMUSP00000027123;ENSMUSP00000119336 | 35 | 34 | Hspd1 | **3,03** | ***** |
|  | **88** | ENSMUSP00000068594;ENSMUSP00000123749 | 21 | 21 | Hyou1 | **-2,09** | ***** |
|  | **89** | ENSMUSP00000050446;ENSMUSP00000094870 | 3 | 2 | Irgm1 | **1,82** | ***** |
|  | **90** | ENSMUSP00000040140;ENSMUSP00000035610 | 11 | 10 | Lman1 | **-1,97** | ***** |
|  | **91** | ENSMUSP00000030986;ENSMUSP00000120233 | 3 | 3 | Lrpap1 | **-4,56** | ***** |
|  | **92** | ENSMUSP00000021239 | 6 | 6 | Lrrc59 | **1,45** | ns |
|  | **93** | ENSMUSP00000046856 | 3 | 3 | Lss | **-1,35** | ***** |
|  | **94** | ENSMUSP00000066534;ENSMUSP00000123907 | 3 | 3 | Manf | **-3,66** | ***** |
|  | **95** | ENSMUSP00000008684;ENSMUSP00000112646 | 12 | 12 | Mgst1 | **1,30** | ns |
|  | **96** | ENSMUSP00000055776;ENSMUSP00000107749 | 2 | 2 | Mlec | **-1,28** | ns |
|  | **97** | ENSMUSP00000032114 | 11 | 11 | Mogs | **-1,72** | ***** |
|  | **98** | ENSMUSP00000029805 | 29 | 28 | Mttp | **-2,47** | ***** |
|  | **99** | ENSMUSP00000070388 | 2 | 2 | Myo1c | **-1,76** | ns |
|  | **100** | ENSMUSP00000020463 | 4 | 3 | Ncln | **1,50** | ***** |
|  | **101** | ENSMUSP00000026222;ENSMUSP00000128192 | 4 | 4 | Ndufb8 | **2,03** | ***** |
|  | **102** | ENSMUSP00000033715 | 3 | 3 | Nsdhl | **-1,36** | ***** |
|  | **103** | ENSMUSP00000026122;ENSMUSP00000128796 | 34 | 32 | P4hb | **-2,98** | ***** |
|  | **104** | ENSMUSP00000028683;ENSMUSP00000119337 | 27 | 26 | Pdia3 | **-2,38** | ***** |
|  | **105** | ENSMUSP00000076521 | 21 | 21 | Pdia4 | **-3,77** | ***** |
|  | **106** | ENSMUSP00000023550 | 10 | 10 | Pdia5 | **-2,11** | ***** |
|  | **107** | ENSMUSP00000052912 | 17 | 16 | Pdia6 | **-3,23** | ***** |
|  | **108** | ENSMUSP00000073061;ENSMUSP00000056643 | 9 | 9 | Pgrmc1 | **-2,11** | ***** |
|  | **109** | ENSMUSP00000005651;ENSMUSP00000112924 | 32 | 31 | Por | **-2,15** | ***** |
|  | **110** | ENSMUSP00000034947 | 14 | 14 | Ppib | **-2,64** | ***** |
|  | **111** | ENSMUSP00000003493 | 5 | 4 | Prkcsh | **-1,66** | ***** |
|  | **112** | ENSMUSP00000028238;ENSMUSP00000003121 | 5 | 4 | Rab14 | **1,40** | ***** |
|  | **113** | ENSMUSP00000082352 | 4 | 4 | Rdh11 | **1,05** | ns |
|  | **114** | ENSMUSP00000039252;ENSMUSP00000076735 | 8 | 4 | Rdh7 | **-1,74** | ***** |
|  | **115** | ENSMUSP00000068568;ENSMUSP00000101140 | 4 | 4 | Retsat | **-1,07** | ns |
|  | **116** | ENSMUSP00000008826;ENSMUSP00000100795 | 4 | 4 | Rpl10 | **4,89** | ***** |
|  | **117** | ENSMUSP00000032143 | 18 | 17 | Rpn1 | **-1,72** | ***** |
|  | **118** | ENSMUSP00000112081;ENSMUSP00000029171 | 15 | 15 | Rpn2 | **-1,51** | ***** |
|  | **119** | ENSMUSP00000016072;ENSMUSP00000040560 | 7 | 6 | Rrbp1 | **-1,09** | ns |
|  | **120** | ENSMUSP00000023453 | 2 | 2 | Sdf2l1 | **-1,78** | ***** |
|  | **121** | ENSMUSP00000026818 | 2 | 2 | Sec11a | **-2,50** | ***** |
|  | **122** | ENSMUSP00000029476;ENSMUSP00000117579 | 2 | 2 | Sec22b | **-2,99** | ***** |
|  | **123** | ENSMUSP00000021375 | 2 | 2 | Sec23a | **1,90** | ***** |
|  | **124** | ENSMUSP00000032168 | 2 | 2 | Sec61a1 | **1,96** | ***** |
|  | **125** | ENSMUSP00000019937 | 3 | 3 | Sec63 | **-1,26** | ns |
|  | **126** | ENSMUSP00000021347 | 2 | 2 | Sel1l | **-1,14** | ns |
|  | **127** | ENSMUSP00000057595;ENSMUSP00000117145 | 22 | 22 | Slc27a2 | **-1,24** | ns |
|  | **128** | ENSMUSP00000032539;ENSMUSP00000112495 | 23 | 22 | Slc27a5 | **-1,22** | ns |
|  | **129** | ENSMUSP00000021864 | 2 | 2 | Ssr1 | **-1,72** | ***** |
|  | **130** | ENSMUSP00000002090 | 2 | 2 | Ssr4 | **-1,37** | ns |
|  | **131** | ENSMUSP00000113116 | 6 | 5 | Stt3a | **-2,52** | ***** |
|  | **132** | ENSMUSP00000015011;ENSMUSP00000125327 | 2 | 2 | Surf4 | **-1,07** | ns |
|  | **133** | ENSMUSP00000019382 | 4 | 4 | Tecr | **-1,21** | ns |
|  | **134** | ENSMUSP00000025713 | 2 | 2 | Tm7sf2 | **-1,81** | ns |
|  | **135** | ENSMUSP00000037583 | 3 | 3 | Tmed10 | **-1,03** | ns |
|  | **136** | ENSMUSP00000041839 | 9 | 9 | Txndc5 | **-2,66** | ***** |
|  | **137** | ENSMUSP00000037930;ENSMUSP00000134078 | 11 | 10 | Uggt1 | **-2,40** | ***** |
|  | **138** | ENSMUSP00000072803;ENSMUSP00000037258 | 27 | 14 | Ugt1a1 | **-1,99** | ***** |
|  | **139** | ENSMUSP00000014263 | 20 | 3 | Ugt1a6a | **-1,49** | ns |
|  | **140** | ENSMUSP00000073444 | 17 | 5 | Ugt1a9 | **-1,74** | ***** |
|  | **141** | ENSMUSP00000024897 | 6 | 5 | Vapa | **-1,30** | ns |
|  | **142** | ENSMUSP00000064699 | 4 | 3 | Vapb | **-1,37** | ***** |
|  | **143** | ENSMUSP00000030164;ENSMUSP00000026486 | 5 | 5 | Vcp | **-2,07** | ns |
